# The Effect of Short-Term Dietary Fructose Supplementation on Gastric Emptying Rate and Gastrointestinal Hormone Responses in Healthy Men

**DOI:** 10.3390/nu9030258

**Published:** 2017-03-10

**Authors:** Adora M. W. Yau, John McLaughlin, Ronald J. Maughan, William Gilmore, Gethin H. Evans

**Affiliations:** 1 School of Healthcare Science, Manchester Metropolitan University, Manchester, Greater Manchester M1 5GD, UK; a.yau@mmu.ac.uk (A.M.W.Y.); b.gilmore@mmu.ac.uk or ws.gilmore@ulster.ac.uk (W.G.); 2 Institute of Inflammation and Repair, Faculty of Medical and Human Sciences, University of Manchester, Manchester, Greater Manchester M13 9PT, UK; john.mclaughlin@manchester.ac.uk; 3 School of Sport, Exercise and Health Sciences, Loughborough University, Loughborough, Leicestershire LE11 3TU, UK; R.J.Maughan@lboro.ac.uk; 4 School of Biomedical Sciences, Ulster University, Cromore Road, Coleraine, Co Londonderry BT52 1SA, UK

**Keywords:** fructose supplementation, glucose, fructose, sugar ingestion, gastric emptying, gastrointestinal adaptation, gastrointestinal hormones

## Abstract

This study aimed to examine gastric emptying rate and gastrointestinal hormone responses to fructose and glucose ingestion following 3 days of dietary fructose supplementation. Using the ^13^C-breath test method, gastric emptying rates of equicaloric fructose and glucose solutions were measured in 10 healthy men with prior fructose supplementation (fructose supplement, FS; glucose supplement, GS) and without prior fructose supplementation (fructose control, FC; glucose control, GC). In addition, circulating concentrations of acylated ghrelin (GHR), glucagon-like peptide-1 (GLP-1), glucose-dependent insulinotropic polypeptide (GIP), and insulin were determined, as well as leptin, lactate, and triglycerides. Increased dietary fructose ingestion resulted in accelerated gastric emptying rate of a fructose solution but not a glucose solution. No differences in GIP, GLP-1, or insulin incremental area under curve (iAUC) were found between control and supplement trials for either fructose or glucose ingestion. However, a trend for lower ghrelin iAUC was observed for FS compared to FC. In addition, a trend of lower GHR concentration was observed at 45 min for FS compared to FC and GHR concentration for GS was greater than GC at 10 min. The accelerated gastric emptying rate of fructose following short-term supplementation with fructose may be partially explained by subtle changes in delayed postprandial ghrelin suppression.

## 1. Introduction

Gastric emptying is a rate-limiting step in the delivery and absorption of nutrients and fluids in the small intestine. Therefore, the rate at which nutrients empty from the stomach directly affects the period of gastric distension and nutrient sensing. Gastric distension causes both satiation and satiety [1] and a prolonged period of gastric distension due to delayed emptying may lead to a prolonged satiety period. A number of hormones secreted from the gastrointestinal tract involved in appetite regulation have also been to shown to influence gastric emptying rate. Ghrelin, the only orexigenic hormone, accelerates gastric emptying rate [2,3] whilst satiety hormones such as glucagon-like peptide-1 (GLP-1), peptide tyrosine tyrosine (PYY), and cholecystokinin (CCK) inhibit gastric emptying rate [4,5,6,7,8]. 

The gastrointestinal tract has been shown to be a highly adaptive organ. Gastric emptying in humans has been shown to be influenced by previous dietary intake. Increases in the gastric emptying rate of a high-fat test meal following three days of a high-fat diet [9] and increases in the gastric emptying rate of a glucose test solution following 3 days of high glucose intake [10,11] have been shown. More recently, three days of dietary fructose supplementation has been shown to result in a monosaccharide specific acceleration of a fructose solution but not a glucose solution [12]. One potential mechanism for this adaptation is an alteration in gastrointestinal hormone response. 

A small number of studies that have investigated the effects of previous dietary intake on gut hormone responses in humans have shown changes in the secretion of gut-derived hormones. Most of this work to date has been conducted on the effects of a high-fat diet, however, and few have simultaneously measured gastric emptying rate. Following the observations by Cunningham et al. [13] where emptying rate of a fatty meal was accelerated as a result of a high-fat diet for two weeks, it was reported that a high-fat diet resulted in an increase in postprandial CCK concentration [14]. Fasting levels of CCK have also been shown to be altered in humans following three weeks of a high-fat diet compared to an isoenergetic low-fat diet [15]. The effect of a high-fat diet has also been shown by others to suppress postprandial ghrelin response to a greater extent [16], but result in unaltered fasting concentration and postprandial response for GLP-1 [17]. 

With regards to increased dietary intake of carbohydrates, increased glucose ingestion for 4–7 days resulted in accelerated gastric emptying of glucose and fructose solutions but differential gut hormones responses [11]. Greater glucose-dependent insulinotropic polypeptide (GIP) hormone responses were observed following the glucose-supplemented diet for both sugar solutions [11]. However, insulin response was greater following glucose ingestion but unchanged following fructose ingestion in the glucose-supplemented trials [11]. In addition, it followed that glycaemic response was lower for glucose ingestion but not for fructose ingestion following glucose supplementation [11]. The only study to our knowledge that has investigated the effects of increased fructose consumption on gut hormones showed that two weeks of a high-fructose diet in rats increased fasting ghrelin levels by 40% [18]. The effect of increased fructose consumption over a shorter period of time on moderations of postprandial gut hormone responses in relation to adaptations of gastric emptying rate in humans is unknown. Therefore, the aim of this study was to investigate the effect of a short-term increase in dietary fructose ingestion on gastric emptying rate and associated gastrointestinal hormone responses in healthy men.

## 2. Materials and Methods

### 2.1. Participants

Ten healthy men (mean ± standard deviation, age 26 ± 7 years, height 179.0 ± 6.3 cm, body mass 81.2 ± 11.1 kg, body mass index 25.3 ± 3.1 kg·m^−2^, and estimated body fat 23.2% ± 8.1%) volunteered to take part in this investigation. All participants were non-smokers, had no history of chronic gastrointestinal disease, were not consuming medication with any known effect on gastrointestinal function and had no medical conditions as assessed by a medical screening questionnaire. The participants provided written informed consent prior to participation and ethical approval was provided via the Institutional Ethical Advisory Committee (Reference: SE111228).

### 2.2. Experimental Protocol

Experimental trials were conducted in a single-blind, randomised, crossover fashion commencing between 0800 and 0900 h following an overnight fast from 2100 h with the exception of drinking 500 mL of water approximately 90 min before arrival at the laboratory. Participants reported to the laboratory on four occasions to complete four experimental trials; fructose with supplementation (FS), fructose with water control (FC), glucose with supplementation (GS) and glucose with water control (GC) as previously conducted by Yau et al. [12]. Experimental trials were separated by a minimum period of 7 days. A 3-day dietary and physical activity maintenance period preceded each experimental trial. Participants were asked to record their diet and physical activity prior to their first trial then to replicate them in the remaining three trials. The purpose of this was to ensure standardisation and consistency of macronutrient intake and metabolic status in the days leading up to each trial. Furthermore, participants were asked to refrain from alcohol and caffeine consumption and the performance of strenuous physical activity in the 24 h preceding each experimental trial. In addition to their normal dietary intake, participants were asked to consume either four 500 mL bottles of water (control trials) or four 500 mL solutions, each containing 30 g fructose (supplement trials) per day over the 3-day dietary maintenance period. Participants were instructed to consume these drinks evenly throughout the day in between meals.

Upon arrival at the laboratory, participants were asked to completely empty their bladder into a container from which a 5 mL urine sample was retained for later analysis of osmolality. Body mass was subsequently recorded before an intravenous cannula was inserted into an antecubital vein. This remained in place for the duration of the experimental trial and was kept patent with infusion of saline after each blood sample collection. A baseline 5 mL blood sample was collected before participants ingested 595 mL of a fructose solution (36 g dissolved in water and prepared to a volume of 600 mL) or an equicaloric glucose monohydrate solution (39.6 g dissolved in water and prepared to a volume of 600 mL). A 5 mL sample of the test solutions was retained for osmolality analysis. Participants were given a maximum of two minutes to consume the test solution and instructed to consume it as quickly as they were able to. Participants remained in a semi-supine position for 60 min following drink ingestion where further blood samples were collected at 10, 20, 30, 45 and 60 min post ingestion. Ratings of appetite (hunger, fullness, prospective food consumption) were assessed at baseline and at 10-min intervals following drink ingestion for 60 min using a 100-mm visual analogue scale (VAS). Following the last sample collection the cannula was removed before participants left the laboratory.

### 2.3. Gastric Emptying Measurement

Gastric emptying was assessed using the ^13^C-acetate breath method as described in Yau et al. [12]. Prior to ingestion of the fructose or glucose test drink containing 100 mg ^13^C-sodium acetate (Cambridge Isotope Laboratories Inc., Andover, MA, USA), a basal end-expiratory breath sample was collected. Further end-expiratory breath samples were collected at 10-min intervals over a period of 60 min following drink ingestion. Breath samples were collected into a 100-mL foil bag on each occasion by exhalation through a mouthpiece. Bags were then sealed with a plastic stopper and stored for later analysis.

Breath samples were analysed by non-dispersive infra-red spectroscopy (IRIS, Wagner Analyzen-Technik, Bremen, Germany) for the ratio of ^13^CO_2_:^12^CO_2_. The differences in the ratio of ^13^CO_2_:^12^CO_2_ from baseline breath to post breath samples are expressed as delta over baseline (DOB). Half emptying time (T_½_) and time of maximum emptying rate (T_lag_) were calculated using the manufacturer’s integrated software evaluation embedded with the equations of Ghoos et al. [19]. Each participant’s own physiological CO_2_ production assumed as 300 mmol CO_2_ per m^2^ body surface per hour was set as default and body surface area was calculated by the integrated software according to the formula of Haycock et al. [20].

### 2.4. Sample Analysis

Urine, drink and serum samples were analysed in duplicate for osmolality by freezing point depression (Gonotec Osmomat 030, Gonotec, Berlin, Germany). To prevent the degradation of acylated ghrelin, 50 µL of Pefabloc (Roche Diagnostics Limited, Burgess Hill, UK) was immediately added to blood samples. Blood samples were centrifuged at 1500× *g* for 15 min at 4 °C and the serum aliquoted and stored at −80 °C until analysis was performed. Serum glucose, lactate, and triglyceride concentrations were determined in duplicate using a clinical chemistry analyser (Randox Daytona, Crumlin, UK). Serum fructose concentration was determined using a colorimetric assay (EnzyChrom™ EFRU-100; BioAssay Systems, Hayward, CA, USA). Circulating concentrations of acylated ghrelin, insulin, GIP, and leptin were determined using multiplex analysis (Luminex 200, Luminex Corporation, Austin, TX, USA) with kits purchased from Merck-Millipore (HMHMAG-34K, Milliplex MAP, Merck Millipore Ltd., Feltham, UK). Circulating concentrations of total GLP-1 were determined in duplicate using Enzyme Linked Immunoassay (EZGLP1T-36K, Merck Millipore Ltd., Feltham, UK).

### 2.5. Data and Statistical Analysis

The trapezoid method was utilised to calculate incremental area under curve (iAUC). Differences in pre-ingestion body mass, pre-ingestion urine osmolality, drink osmolality and iAUC for serum blood measures were examined using one-way repeated analysis of variance (ANOVA). Post-hoc analysis consisted of Bonferroni adjusted pairwise comparisons. Two-way repeated ANOVA were used to examine differences in gastric emptying DOB values, serum blood measures, and subjective appetite VAS scores. Sphericity for repeated measures was assessed, and where appropriate, Greenhouse–Geisser corrections were applied for epsilon <0.75, and the Huynh–Feldt correction adopted for less severe asphericity. Significant *F*-tests were followed by dependent Student’s *t*-Tests or one-way repeated ANOVA and Bonferroni adjusted pairwise comparisons as appropriate. Gastric emptying T_½_ and T_lag_ data were examined with dependent Student’s *t*-Tests to test the hypothesis of interest (i.e., effect of supplementation on gastric emptying rate of fructose and of glucose). All data were analysed using SPSS Statistics for Windows version 21 (IBM, New York, NY, USA). Statistical significance was accepted at the 5% level and results presented as means and standard deviations.

## 3. Results

### 3.1. Body Mass, Hydration Status and Drink Osmolality

Body mass (Table 1) remained stable over the duration of the study (*p* = 0.338). Pre-ingestion urine osmolality (Table 1) was generally lower in each supplement trial compared to the control trials but was not statistically significant (*p* = 0.067). Drink osmolalities were 368 ± 3, 367 ± 4, 371 ± 3 and 370 ± 4 mOsmol/kg (*p* = 0.010) for FC, FS, GC and GS, respectively. Post hoc analysis indicated no significant differences between trials.

### 3.2. Gastric Emptying

**Figure 1:**
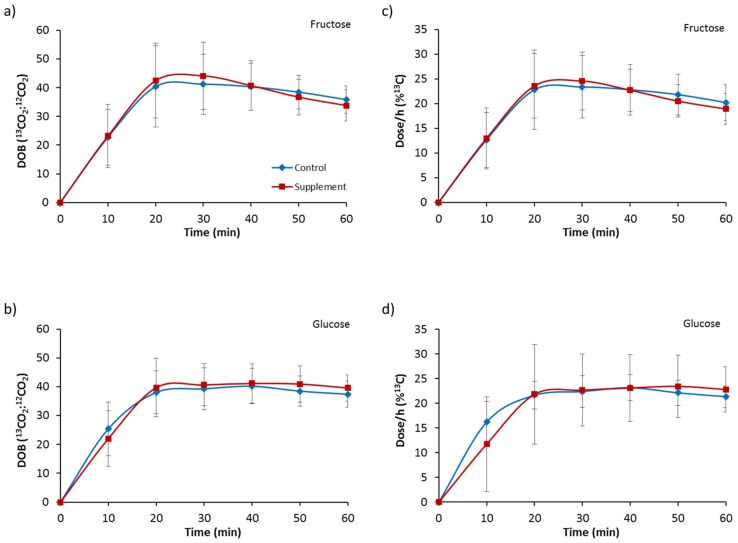
Gastric emptying delta over baseline (DOB) for 60 min following ingestion of (**a**) a 6% fructose solution and (**b**) a 6% glucose solution, and dose/h (%^13^C) following ingestion of (**c**) a 6% fructose solution and (**d**) a 6% glucose solution. Treatments were control without fructose supplementation and with three days of supplementation with 120 g fructose per day. Values are mean ± standard deviation.

Gastric emptying T_½_ for fructose ingestion was accelerated after the period of dietary supplementation with fructose compared to the control (FC, 59 ± 13 min vs. FS, 51 ± 10 min; *p* = 0.004). The same was also observed for T_lag_, with dietary fructose supplementation accelerating fructose ingestion T_lag_ (FC, 37 ± 3 min vs. FS, 32 ± 7 min; *p* = 0.026). In contrast, gastric emptying T_½_ for glucose ingestion did not change with fructose supplementation (GC, 75 ± 18 min vs. GS, 68 ± 16 min; *p* = 0.245), and neither did T_lag_ (GC, 38 ± 7 min vs. GS, 40 ± 7 min; *p* = 0.679). Breath DOB values for fructose ingestion (Figure 1a) revealed no main effect of trial (*p* = 0.912), a significant main effect of time (*p* < 0.001) and no interaction effect (*p* = 0.376). The ratio of ^13^CO_2_ to ^12^CO_2_ was significantly increased at all post-ingestion time-points compared to baseline and 10 min (*p* < 0.01) for FC. The ratio of ^13^CO_2_ to ^12^CO_2_ was significantly increased at all post-ingestion time-points compared to baseline (*p* < 0.01) and from 20 to 40 min compared to 10 min (*p* < 0.01) for FS. Breath DOB for glucose ingestion (Figure 1b) showed no main effect of trial (*p* = 0.537), a significant main effect of time (*p* < 0.001) and no interaction effect (*p* = 0.282). The ratio of ^13^CO_2_ to ^12^CO_2_ was significantly increased at all post-ingestion time-points compared to baseline and at 10 min (*p* < 0.01) for GC and GS. Data for dose/h (%^13^C) are provided in Figure 1c,d.

### 3.3. Gut Hormones

#### 3.3.1. Ghrelin

**Figure 2:**
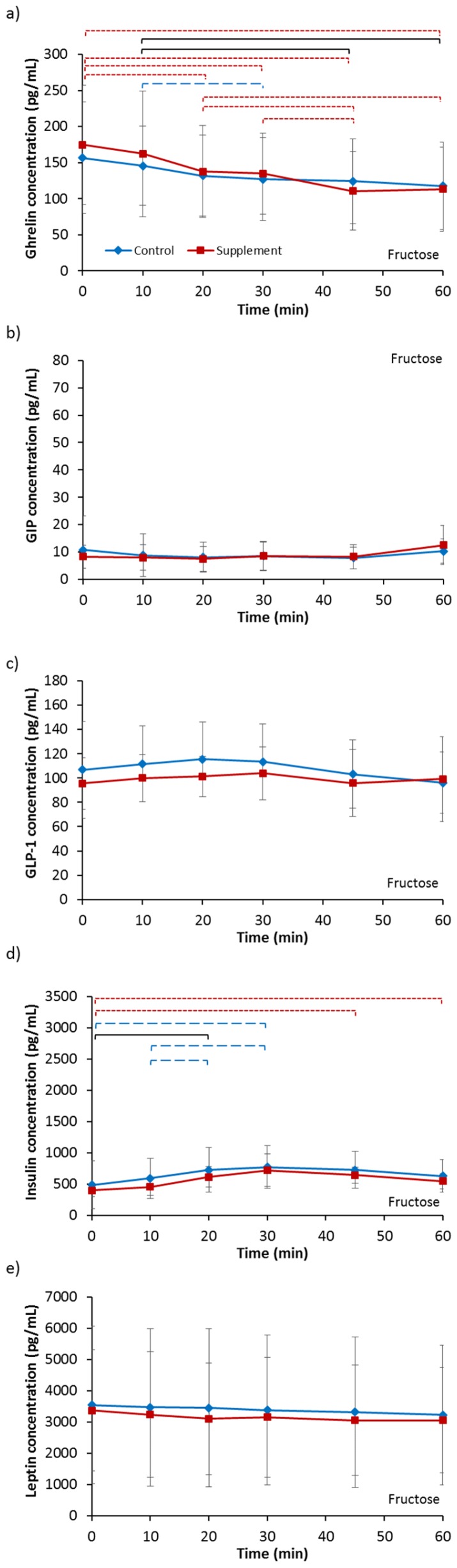
Serum concentrations of (**a**) ghrelin; (**b**) glucose-dependent insulinotropic polypeptide (GIP); (**c**) glucagon-like peptide-1 (GLP-1); (**d**) insulin and (**e**) leptin for 60 min following ingestion of a 6% fructose solution. Treatments were control without fructose supplementation and with three days of supplementation with 120 g fructose per day. Brackets denote significant difference between time-points, blue long dashed for control trial only, red small dashed for supplement trial only and black solid for both trials (*p <* 0.05). Values are mean ± standard deviation.

**Figure 3:**
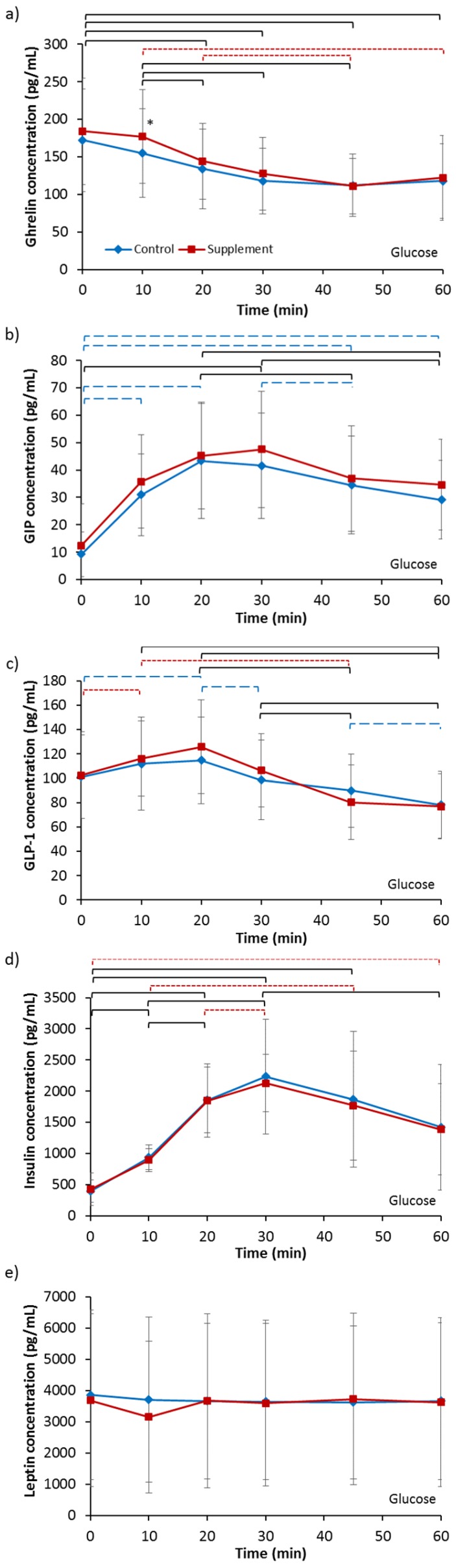
Serum concentrations of (**a**) ghrelin; (**b**) GIP; (**c**) GLP-1; (**d**) insulin and (**e**) leptin for 60 min following ingestion of a 6% glucose solution. Treatments were control without fructose supplementation and with three days of supplementation with 120 g fructose per day. * Significantly greater for supplement compared to control (*p <* 0.05). Brackets denote significant difference between time-points, blue long dashed for control trial only, red small dashed for supplement trial only and black solid for both trials (*p <* 0.05). Values are mean ± standard deviation.

Baseline ghrelin concentrations (Table 2) were not different between any of the four trials (*p* = 0.131). However, there was a pattern for higher baseline levels following supplementation compared to each respective control trial. For fructose ingestion, this tended to significance (*p* = 0.089). Analysis of fructose ingestion (Figure 2a) revealed no main effect of supplementation (*p* = 0.264) but an effect of time (*p* < 0.001) and a trend of an interaction (*p* = 0.065). Post-hoc analysis revealed that ghrelin concentration significantly decreased between 10 min and 60 min in FC whilst the decrease from baseline levels in FS occurred from 20 min after ingestion. A trend of lower ghrelin concentration was also indicated for FS compared to FC at 45 min after ingestion (*p* = 0.063). There was a trend in a lower iAUC for FS compared to FC (FC, −1506.94 ± 1704.50 pg/mL vs. FS, −2514.09 ± 1151.33 pg/mL; *p* = 0.053). Analysis for glucose ingestion (Figure 3a) revealed a trend of a supplementation effect (*p* = 0.080), an effect of time (*p <* 0.001) and no interaction effect (*p* = 0.276). Post-hoc analysis showed ghrelin concentration significantly decreased from baseline levels at 20 min to 60 min after ingestion in both GC and GS. Furthermore, ghrelin concentration was significantly higher in GS compared to GC at 10 min after ingestion (*p* = 0.019). There was no difference in iAUC between GC and GS (GC, −2535.20 ± 1530.65 pg/mL vs. GS, −2826.96 ± 1499.31 pg/mL; *p* = 0.478).

#### 3.3.2. GIP

Baseline GIP concentrations (Table 2) were not different between any of the four trials (*p* = 0.545). Analysis for fructose ingestion (Figure 2b) showed no effect of supplementation (*p* = 0.760), time (*p* = 0.121) or interaction (*p* = 0.368). There was no difference in iAUC between FC and FS (FC, −124.98 ± 435.74 pg/mL vs. FS, 22.04 ± 169.69 pg/mL; *p* = 0.346). Analysis for glucose ingestion (Figure 3b) revealed a trend of a supplementation effect (*p* = 0.076), a main effect of time (*p* < 0.001) but no interaction effect (*p* = 0.707). GIP concentration for GC significantly increased from baseline values by 10 min then decreased from 20 min but remained significantly higher than baseline at 60 min. GIP concentration for GS, on the other hand, significantly increased from baseline at 30 min and remained elevated from baseline at 60 min but not significantly. There was no difference in iAUC between GS and GC (GC, 1485.26 ± 644.97 pg/mL vs. GS, 1518.83 ± 1275.25 pg/mL; *p* = 0.911). 

#### 3.3.3. GLP-1

Baseline GLP-1 concentrations (Table 2) were not different between any of the four trials (*p* = 0.719). Analysis for fructose ingestion (Figure 2c) showed no main effect of supplementation (*p* = 0.339), an effect of time tending to significance (*p* = 0.081) and no interaction effect (*p* = 0.328). No difference in iAUC was seen between FC and FS (FC, −80.46 ± 142.16 pg/mL vs. FS, 27.70 ± 142.48 pg/mL; *p* = 0.178). Analysis for glucose ingestion (Figure 3c) showed no main effect of supplementation (*p* = 0.747), an effect of time (*p <* 0.001) and an interaction effect tending to significance (*p* = 0.064). Post hoc analysis revealed GLP-1 concentration increased significantly from baseline at 20 min then decreased significantly at every time-point in GC. For GS, concentrations significantly increased from baseline at 10 min, then increased further non-significantly at 20 min before significantly decreasing. No difference in iAUC was observed (GC, −152.36 ± 667.66 pg/mL vs. 100.16 ± 908.36 pg/mL; *p* = 0.492).

#### 3.3.4. Insulin

Baseline insulin concentrations (Table 2) were not different between any of the four trials (*p* = 0.750). Analysis for fructose ingestion (Figure 2d) showed no main effect of supplementation (*p* = 0.341), an effect of time (*p <* 0.001) and no interaction effect (*p* = 0.778). Post hoc analysis showed a significant increase in insulin from baseline levels for both FC and FS. No difference in iAUC was observed between FC and FS (FC, 1079.96 ± 2019.57 pg/mL vs. FS, 1109.93 ± 793.25 pg/mL; *p* = 0.958). Analysis for glucose ingestion (Figure 3d) showed no main effect of supplementation (*p* = 0.975), an effect of time (*p* < 0.001) and no interaction effect (*p* = 0.844). Post hoc analysis showed insulin concentrations increased significantly from baseline values at 30 min then significantly decreased thereafter for both GC and GS. No difference in iAUC was present (GC, 72,133.17 ± 32,863.68 pg/mL vs. GS, 68,512.93 ± 15,821.44 pg/mL; *p* = 0.626).

#### 3.3.5. Leptin

Baseline leptin concentrations (Table 2) were not different between any of the four trials (*p* = 0.484). Analysis for fructose ingestion (Figure 2e) showed no effect of supplementation (*p* = 0.302), time (*p* = 0.100) or interaction (*p* = 0.466). No difference in iAUC between FC and FS was present (FC, −2389.11 ± 3623.58 pg/mL vs. FS, −2387.77 ± 3522.92 pg/mL; *p* = 0.999). Analysis for glucose ingestion (Figure 3e) also showed no effect of supplementation (*p* = 0.934), time (*p* = 0.378) or interaction (*p* = 0.294. No difference in iAUC was present between GC and GS (GC, −10,592.99 ± 13,423.93 pg/mL vs. GS, −3557.61 ± 10,977.20 pg/mL; *p* = 0.147).

### 3.4. Blood Glucose and Fructose

**Figure 4:**
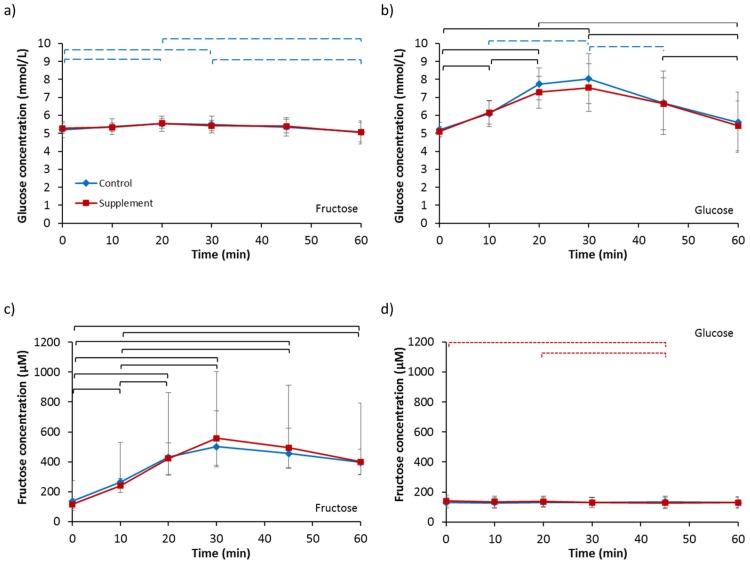
Serum concentrations of glucose for 60 min following ingestion of (**a**) a 6% fructose solution and (**b**) a 6% glucose solution and serum concentrations of fructose following ingestion of (**c**) a 6% fructose solution and (**d**) a 6% glucose solution. Treatments were control without fructose supplementation and with three days of supplementation with 120 g fructose per day. Brackets denote significant difference between time-points, blue long dashed for control trial only, red small dashed for supplement trial only and black solid for both trials (*p <* 0.05). Values are mean ± standard deviation.

Baseline serum glucose concentrations (Table 2) were not different between any of the four trials (*p* = 0.591). Analysis for fructose ingestion (Figure 4a) revealed no effect of supplementation (*p* = 0.880), an effect of time (*p* = 0.024) and no interaction effect (*p* = 0.928). Post-hoc analysis showed serum glucose concentration significantly increased at 20 min from baseline concentrations then decreased significantly at 60 min for FC. A similar response pattern over time for FS was not significantly different (*p* = 0.174). No difference in iAUC was observed between FC and FS (FC, 9.75 ± 5.39 mmol/L vs. FS, 5.53 ± 19.02 mmol/L; *p* = 0.438). Analysis for glucose ingestion (Figure 4b) showed no effect of supplementation (*p* = 0.428), an effect of time (*p <* 0.001) and no interaction effect (*p* = 0.658). Post hoc analysis revealed serum glucose concentrations significantly increased from baseline, peaking at 30 min, and then decreased significantly to near baseline levels at 60 min for both GC and GS. No difference in iAUC existed (GC, 95.07 ± 56.21 mmol/L vs. GS, 88.17 ± 54.12 mmol/L; *p* = 0.711).

Baseline serum fructose concentrations (Table 2) were not different between any of the four trials (*p* = 0.163). Analysis for fructose ingestion (Figure 4c) showed no main effect of supplementation (*p* = 0.948), an effect of time (*p <* 0.001) and an interaction effect (*p* = 0.011). Post hoc analysis revealed serum fructose concentrations increased rapidly from baseline concentrations within the first 10 min for both FC and FS. There was a strong tendency for iAUC to be higher in FS compared to FC (FC, 15,505.00 ± 4377.39 µM vs. FS, 17,583.45 ± 4597.19 µM; *p* = 0.050). Analysis for glucose ingestion (Figure 4d) showed no main effect of supplementation (*p* = 0.547), no effect of time (*p* = 0.172) but an interaction effect (*p* = 0.036). Post-hoc analysis revealed serum fructose concentrations did not change over time in GC (*p* = 0.645), but in GS, concentrations were significantly lower at 45 min compared to baseline (*p* = 0.041) and 20 min (*p* = 0.017). No difference in iAUC was observed (GC, 41.73 ± 889.46 µM vs. GS, −409.76 ± 457.67 µM; *p* = 0.226).

### 3.5. Lactate and Triglycerides

**Figure 5:**
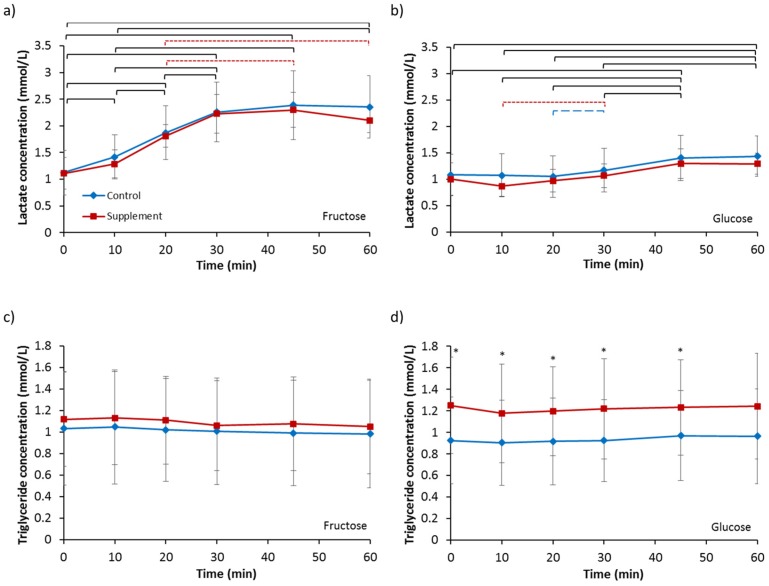
Serum concentrations of lactate following ingestion of (**a**) a 6% fructose solution and (**b**) a 6% glucose solution and serum concentrations of triglyceride following ingestion of (**c**) a 6% fructose solution and (**d**) a 6% glucose solution. Treatments were control without fructose supplementation and with three days of supplementation with 120 g fructose per day. Brackets denote significant difference between time-points, blue long dashed for control trial only, red small dashed for supplement trial only and black solid for both trials (*p* < 0.05). * Significantly greater for supplement compared to control (*p* < 0.05). Values are mean ± standard deviation.

Baseline serum lactate concentrations (Table 2) were not different between any of the four trials (*p* = 0.686). Analysis for fructose ingestion (Figure 5a) revealed no effect of supplementation (*p* = 0.511), an effect of time (*p <* 0.001) and no interaction effect (*p* = 0.457). Lactate concentrations increased significantly from baseline values from 10 min for both FC and FS. No difference in iAUC was observed (FC, 51.63 ± 22.84 mmol/L vs. FS, 47.86 ± 15.17 mmol/L; *p* = 0.482). Analysis for glucose ingestion (Figure 5b) showed no effect of supplementation (*p* = 0.198), an effect of time (*p <* 0.001) and no interaction effect (*p* = 0.621). Lactate concentrations increased significantly from 45 min onwards compared to baseline for both GC and GS. No difference in iAUC was observed (GC, 8.14 ± 7.84 mmol/L vs. 6.30 ± 10.65 mmol/L; *p* = 0.331).

Baseline triglyceride concentrations (Table 2) were not different between any of the four trials (*p* = 0.082). Analysis for fructose ingestion (Figure 5c) revealed no effect of supplementation (*p* = 0.944), a trend for an effect of time (*p* = 0.069) and no interaction effect (*p* = 0.726). No difference in iAUC was seen (FC, −1.31 ± 5.01 mmol/L vs. FS, −1.30 ± 5.21 mmol/L; *p* = 0.998). Analysis for glucose ingestion (Figure 5d) showed a main effect of supplementation (*p* = 0.021), but no significant effect of time (*p* = 0.287) or interaction (*p* = 0.596). Triglyceride concentration was significantly greater for GS compared to GC at all time points (*p* < 0.05) except at 60 min where it was strongly tending to significance (*p* = 0.051). Incremental AUC was not different between GC and GS (GC, 0.69 ± 2.45 mmol/L vs. GS, −1.49 ± 5.55 mmol/L; *p* = 0.243).

### 3.6. Appetite Ratings

**Figure 6:**
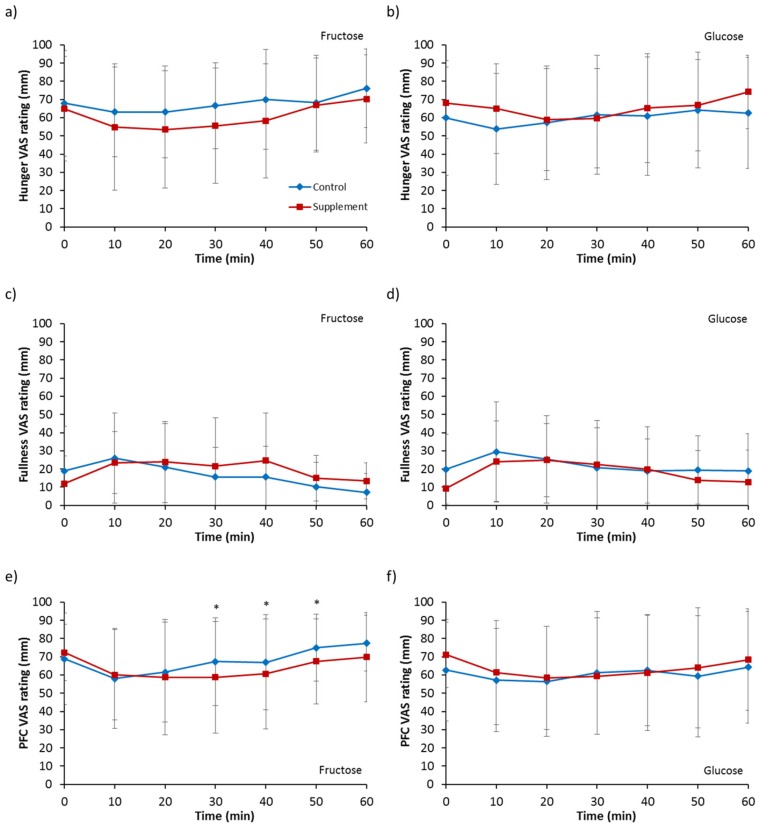
Visual analogue scale (VAS) appetite ratings of hunger following ingestion of (**a**) a 6% fructose solution and (**b**) a 6% glucose solution, ratings of fullness following ingestion of (**c**) a 6% fructose solution and (**d**) a 6% glucose solution, and ratings of prospective food consumption (PFC) following ingestion of (**e**) a 6% fructose solution and (**f**) a 6% glucose solution. Treatments were control without fructose supplementation and with three days of supplementation of 120 g fructose per day. * Significantly greater for control trial compared to supplement (*p* < 0.05). Values are mean ± standard deviation.

Hunger ratings for fructose ingestion (Figure 6a) showed a trend of a supplementation effect (*p* = 0.090), and no main effect of time (*p* = 0.106) or interaction (*p* = 0.477). Ingestion of a glucose solution (Figure 6b) also showed no main effect of supplementation (*p* = 0.231), time (*p* = 0.410) or interaction (*p* = 0.237).

Analysis on feeling of fullness for fructose ingestion (Figure 6c) showed no effect of supplementation (*p* = 0.231), time (*p* = 0.144) or interaction (*p* = 0.236). For glucose ingestion (Figure 6d), a trend of a main effect of supplementation was observed for fullness (*p* = 0.083) but no main effect of time (*p* = 0.235) or interaction (*p* = 0.523). 

No main effect of time (*p* = 0.101) or interaction (*p* = 0.205) was seen for prospective food consumption with fructose ingestion (Figure 6e) but a main effect of supplementation was present (*p* = 0.027). Post hoc analysis revealed ratings were temporarily lower for FS compared to FC from 30 to 50 min. For glucose ingestion (Figure 6f), no effect of supplementation (*p* = 0.550), time (*p* = 0.370) or interaction (*p* = 0.661) was observed.

## 4. Discussion

The gastric emptying results of this study are in agreement to previous findings showing a monosaccharide-specific adaptation in gastric emptying rate following short-term dietary supplementation of fructose [12]. Gastric emptying rate of a solution containing 36 g of fructose was accelerated whilst emptying rate of an equicaloric glucose solution was unchanged. These results may be partially explained by subtle changes in gut hormone responses seen in this present study. Whilst a larger sugar load, such as the typically used load of 75 g, would have resulted in more pronounced effects on secretory endocrine and enteroendocrine hormone responses, these modest loads were utilized in the present study to reflect more commonly ingested amounts of sugar ingestion.

Supplementation of the diet with fructose for three days resulted in a short delay in the postprandial suppression of ghrelin following the ingestion of a fructose solution and a greater ghrelin concentration at 10 min with the ingestion of a glucose solution. Although not significantly different, fasting ghrelin levels were also slightly elevated by 7%–11% after the supplementation period. This is in agreement, proportionally, with the results of Lindqvist et al. [18] who reported a 40% increase in fasting ghrelin concentrations following two weeks of a high-fructose diet in rats. Since ghrelin is known to accelerate gastric emptying rate [2,3], these fasting and postprandial observations would suggest a slight initial acceleration of emptying rate for both fructose and glucose solution ingestion. Therefore, this does not explain the specific acceleration of fructose emptying rate only. However, the differences in the other hormone responses to counter the changes in ghrelin response may offer some explanation. 

One potential explanation is that there was no difference in GIP response for fructose ingestion whilst there was a trend for significantly greater GIP response for glucose ingestion following supplementation. This difference in supplementation effect may have been because GIP secretion is comparatively limited in response to fructose ingestion as seen in the present study and as reported by Kuhre et al. [21] who used a much greater amount of fructose at 75 g. These results contrast those of Horowitz et al. [11] who showed GIP response increased for both glucose and fructose ingestion following dietary glucose supplementation. However, whether these GIP results in the present study indicate a potential mechanism for the specific acceleration of fructose but not glucose emptying is questionable as the influence of GIP on gastric emptying rate is unclear with mixed results. Administration of pharmacological doses of GIP in healthy men have been shown to have no effect on gastric emptying rate [22] as well as moderately accelerating emptying [4]. It may be, therefore, the differences observed in GLP-1 response between fructose ingestion and glucose ingestion that hold the key to the specific gastrointestinal adaptation results. The ingestion of a fructose solution resulted in no significant changes over time in circulating GLP-1 concentration, and although not significantly different, lower concentrations were seen for the supplement trial with a difference tending to significance at 20 min. In addition to greater responses as a result of glucose ingestion compared to fructose ingestion, faster elevations in GLP-1 concentration was observed in the supplement trial compared to the control trial. However, the overall response to glucose ingestion seen in the present study was lower than other studies such as Kuhre et al. [21]. This is likely due to the smaller quantity of sugar ingested in this study compared to 75 g utilised by others. As GLP-1 is known to strongly inhibit gastric emptying and has been termed as an “ileal brake” [4,5,23], this would suggest a greater ileal brake effect to counter the ghrelin increases for glucose ingestion but not for fructose ingestion, resulting in faster fructose emptying rate. Alternatively, other gut hormones not measured in this study may play a more important role. A further potential limitation of the hormones included in the present study is that total GLP-1 and not the active form GLP-1^7−36amide^ was measured. 

Changes in CCK and ghrelin concentrations following high protein or high fat diets have previously been shown to be associated with complementary changes in mRNA levels [24,25]. It is unknown whether any changes in circulating concentrations of gut hormones in this present study were simply changes in hormone release and intestinal feedback or whether the three days of increased dietary fructose load led to up- or down-regulation of genes and associated changes in mRNA levels leading to increased hormone production. This should be investigated further. In terms of the potential mechanism of altered hormone release and intestinal feedback, the increased consumption of fructose may have led to changes in the sensitivity or stimulation to the presence of fructose. This may have been through increased expression of gut sweet taste receptors T1R2/T1R3 which have been detected in the intestinal tract and enteroendocrine cells [26,27] and may potentially be involved in the secretion of gut hormones [28]. It is noted, however, that ingested solutions were not considered to be equisweet and, consequently, activation of sweet taste receptors in the tongue and intestine may have been different between solutions. Equicaloric doses of fructose and glucose were favoured in this study in order to avoid any potential effects of energy density on gastric emptying rate as well as potential effects of different caloric intakes on the secretion of gut-derived hormone response. Alternatively, enhanced absorption of fructose as a result of glucose transporter 5 (GLUT5) up-regulation and consequently greater transporter activity may be involved in the mediation of gut hormone release. 

Three days of fructose supplementation did not result in a change in leptin concentration in this study. This is most likely because there was no change in body mass and thus assumed no change in body fat/adiposity occurred over this short study period where only an extra 1440 kcal was consumed over the three days. This result is in contrast to the results of Le et al. [29] who reported a significant increase in leptin levels within one week of a high-fructose diet. The longer supplementation period with an approximate mean extra 2898 kcal consumption may have accounted for this difference. However, the authors of that study also reported no change in body weight and body fat percentage.

The rate of gastric emptying is expected to have an important impact on the magnitude of both glycaemic and insulinaemic responses. Despite the faster emptying rate, however, serum glucose response to fructose ingestion was not different after supplementation. This suggests that the capacity to metabolise fructose into glucose is not altered and is further supported with the observation that there were no differences in lactate concentration, suggesting that lactate production was also unaltered. Alternatively, greater uptake of glucose by cells may have occurred, though this may be unlikely as no differences were seen for insulin secretion for either fructose or glucose ingestion despite slight variations in incretin hormone responses. The faster gastric emptying of fructose did result in a slightly higher, albeit insignificant, peak serum fructose concentration at 30 min, however. The implications of this, if any, are unknown at this stage. 

Triglyceride concentration was significantly elevated at baseline and remained elevated at all postprandial time-points for glucose ingestion following fructose supplementation. However, no difference was found between the fructose ingestion trials. Taking the glucose ingestion results alone extends the observations that increased fructose intake for seven days can cause significant increases in fasting triglyceride levels [30]. These levels were still far from dyslipidaemia values, however. It is uncertain as to why no differences were also evident at baseline between the fructose control and fructose supplement trials. The results of this short-term feeding study, therefore, do not suggest a link between excessive fructose intake and metabolic dysfunction. It is possible that some of the observations seen by others in more chronic feeding studies, such as elevated fasting concentrations of low density lipoprotein , glucose and insulin [31,32], and decreased insulin sensitivity [32] could be related to changes in gastric emptying rate and gut hormone responses measured in this present study. However, as the present study involved only three days of dietary supplementation, it is difficult to extrapolate the results of the present study to those aforementioned and longer-term studies are required. 

The accelerated emptying of fructose resulted in a trend of greater hunger suppression. It is unlikely that this was due to the hormones studied in the present study as greater ghrelin concentrations are inconsistent with the observed hunger effects. A greater length of exposure of the intestine to fructose may have resulted in greater release of other hormones not measured in the present study that are known to decrease appetite, such as PYY and CCK. In line with the lesser feelings of hunger, lower prospective food consumption was also observed with fructose ingestion following supplementation. The satiety effects of fructose ingestion were therefore greater following increased dietary intake of fructose. The absence of differences in appetite measures with glucose ingestion suggests gastric emptying is an important modulatory process linked to appetite. Whether these changes in subjective feelings of appetite translate to changes in food intake need to be investigated further. 

## 5. Conclusions

In conclusion, the results of this study show that three days of dietary supplementation with 120 g fructose per day results in an accelerated gastric emptying rate of a fructose solution but not a glucose solution. This monosaccharide specific adaptation may be partly explained by moderations of ghrelin secretion, though larger participant numbers may be required to elucidate clearer differences in gut-derived hormone responses following supplementation. The adaptability of the gut and the mechanisms responsible for this should be further investigated with both short- and longer-term studies, along with the subsequent effects on food intake. 

## Figures and Tables

**Table 1:** Pre-trial body mass and urine osmolality as a marker of hydration status.

	FC		FS		GC		GS	
	Mean	SD	Mean	SD	Mean	SD	Mean	SD
Body mass (kg)	80.87	11.15	81.13	11.04	81.48	11.46	80.95	10.80
Urine osmolality (mOsmol/kg)	560	262	397	271	504	266	356	193

FC, fructose ingestion control trial; FS, fructose ingestion supplement trial; GC, glucose ingestion control trial; GS, glucose ingestion supplement trial; SD, standard deviation.

**Table 2:** Baseline concentrations for blood serum measures.

	FC		FS		GC		GS	
	Mean	SD	Mean	SD	Mean	SD	Mean	SD
Ghrelin (pg/mL)	156.65	77.25	174.64	82.47	172.06	68.19	184.05	71.00
GIP (pg/mL)	10.78	12.44	8.26	4.13	9.31	8.18	12.47	15.20
GLP-1 (pg/mL)	106.78	40.07	95.49	21.29	101.12	34.41	102.55	35.53
Insulin (pg/mL)	438.05	383.64	396.53	93.16	396.20	180.37	425.87	260.60
Leptin (pg/mL)	3542.36	2525.04	3371.53	1934.44	3857.64	2711.35	3687.42	2767.98
Glucose (mmol/L)	5.21	0.46	5.28	0.30	5.21	0.40	5.11	0.25
Fructose (µM)	137.0	48.8	115.8	39.6	129.8	36.6	139.4	38.4
Lactate (mmol/L)	1.12	0.41	1.11	0.30	1.08	0.39	1.00	0.30
Triglycerides (mmol/L)	1.03	0.53	1.12	0.44	0.92	0.40	1.25	0.45

FC, fructose ingestion control trial; FS, fructose ingestion supplement trial; GC, glucose ingestion control trial; GS, glucose ingestion supplement trial; SD, standard deviation.

## References

[1] Geliebter A., Westreich S., Gage D. (1988). Gastric distension and gastric capacity in relation to food intake in humans. Physiol. Behav..

[2] Falken Y., Webb D.-L., Abraham-Nordling M., Kressner U., Hellstrom PM., Naslund E. (2013). Intravenous ghrelin accelerates postoperative gastric emptying and time to first bowel movement in humans. Neurogastroent. Motil..

[3] Levin F., Edholm T., Schmidt P.T., Gryback P., Jacobsson H., Degerblad M., Hoybye C., Holst J.J., Rehfeld J.F., Hellstrom P.M. (2006). Ghrelin stimulates gastric emptying and hunger in normal-weight humans. J. Clin. Endocrin. Metab..

[4] Edholm T., Degerblad M., Gryback P., Hilsted L., Holst J.J., Jacobsson H., Efendic S., Schmidt P.T., Hellstrom P.M. (2010). Differential incretin effects of GIP and GLP-1 on gastric emptying, appetite, and insulin-glucose homeostasis. Neurogastroent. Motil..

[5] Wettergren A., Schjoldager B., Mortensen P.E., Myhre J., Christiansen J., Holst J.J. (1993). Truncated glp-1 (proglucagon 78-107-amide) inhibits gastric and pancreatic functions in man. Digest. Dis. Sci..

[6] Witte A.-B., Gryback P., Holst J.J., Hilsted L., Hellstrom P.M., Jacobsson H., Schmidt P.T. (2009). Differential effect of PYY1-36 and PYY3-36 on gastric emptying in man. Regul. Peptides.

[7] Schwizer W., Borovicka J., Kunz P., Fraser R., Kreiss C., D’Amato M., Crelier G., Boesiger P., Fried M. (1997). Role of cholecystokinin in the regulation of liquid gastric emptying and gastric motility in humans: Studies with the CCK antagonist loxiglumide. Gut.

[8] Liddle R.A., Morita E.T., Conrad C.K., Williams J.A. (1986). Regulation of gastric emptying in humans by cholescystokinin. J. Clin. Investig..

[9] Clegg M.E., McKenna P., McClean C., Dabison G.W., Trinick T., Duly E., Shafat A. (2011). Gastrointestinal transit, post-prandial lipaemia and satiety following 3 days high-fat diet in men. Eur. J. Clin. Nutr..

[10] Cunningham K.M., Horowitz M., Read N.W. (1991). The effect of short-term dietary supplementation with glucose on gastric-emptying in humans. Br. J. Nutr..

[11] Horowitz M., Cunningham K.M., Wishart J.M., Jones K.L., Read N.W. (1996). The effect of short-term dietary supplementation with glucose on gastric emptying of glucose and fructose and oral glucose tolerance in normal subjects. Diabetologia.

[12] Yau A.M.W., McLaughlin J., Maughan R.J., Gilmore W., Evans G.H. (2014). Short-term dietary supplementation with fructose accelerates gastric emptying of a fructose but not a glucose solution. Nutrition.

[13] Cunningham K.M., Daly J., Horowitz M., Read N.W. (1991). Gastrointestinal adaptation to diets of differing fat composition in human volunteers. Gut.

[14] French S.J., Murray B., Rumsey R.D.E., Fadzlin R., Read N.W. (1995). Adaptation to high-fat diets—Effects on eating behavior and plasma cholecystokinin. Br. J. Nutr..

[15] Little T.J., Feltrin K.L., Horowitz M., Meyer J.H., Wishart J., Chapman I.M., Feinle-Bisset C. (2007). A high-fat diet raises fasting plasma CCK but does not affect upper gut motility, PYY, and ghrelin, or energy intake during CCK-8 infusion in lean men. Am. J. Physiol. Regul. Integr. Comp. Physiol..

[16] Robertson M.D., Henderson R.A., Vist G.E., Rumsey R.D. (2004). Plasma ghrelin response following a period of acute overfeeding in normal weight men. Int. J. Obes..

[17] Boyd K.A., O’Donovan D.G., Doran S., Wishart J., Chapman I.M., Horowitz M., Feinle C. (2003). High-fat diet effects on gut motility, hormone, and appetite responses to duodenal lipid in healthy men. Am. J. Physiol. Gastrointest. Liver.

[18] Lindqvist A., Baelemans A., Erlanson-Albertsson C. (2008). Effects of sucrose, glucose and fructose on peripheral and central appetite signals. Regul. Pept..

[19] Ghoos Y.F., Maes B.D., Geypens B.J., Mys C., Hiele M.I., Rutgeerts P.J., Vantrappen G. (1993). Measurement of gastric-emptying rate of solids by means of a carbon-labeled octanoic-acid breath test. Gastroenterology.

[20] Haycock G.B., Schwartz G.J., Wisotsky D.H. (1978). Geometric method for measuring body surface area: A height-weight formula validated in infants, children, and adults. J. Pediatr..

[21] Kuhre R.E., Gribble F.M., Hartmann B., Reimann F., Windeløv J.A., Rehfeld J.F., Holst J.J. (2014). Fructose stimulates GLP-1 but not GIP secretion in mice, rats, and humans. Am. J. Physiol. Gastrointest. Liver Physiol..

[22] Meier J.J., Goetze O., Anstipp J., Hagemann D., Holst J.J., Schmidt W.E., Gallwitz B., Nauck M.A. (2004). Gastric inhibitory polypeptide does not inhibit gastric emptying in humans. Am. J. Physiol. Endocrinol. Metab..

[23] Wishart J.M., Horowitz M., Morris H.A., Jones K.L., Nauck M.A. (1998). Relation between gastric emptying of glucose and plasma concentrations of glucagon-like peptide-1. Peptides.

[24] Lee H.M., Wang G.Y., Englander E.W., Kojima M., Greeley G.H. (2002). Ghrelin, a new gastrointestinal endocrine peptide that stimulates insulin secretion: Enteric distribution, ontogeny, influence of endocrine, and dietary manipulations. Endocrinology.

[25] Liddle R.A., Carter J.D., McDonald A.R. (1988). Dietary regulation of rat intestinal cholecystokinin gene expression. J. Clin. Investig..

[26] Bezencon C., le Coutre J., Damak S. (2007). Taste-signaling proteins are coexpressed in solitary intestinal epithelial cells. Chem. Senses.

[27] Dyer J., Salmon K.S.H., Zibrik L., Shirazi-Beechey S.P. (2005). Expression of sweet taste receptors of the T1R family in the intestinal tract and enteroendocrine cells. Biochem. Soc. Trans..

[28] Gerspach A.C., Steinert R.E., Schonenberger L., Graber-Maier A., Beglinger C. (2011). The role of the gut sweet taste receptor in regulating GLP-1, PYY, and CCK release in humans. Am. J. Physiol. Endocrinol. Metab..

[29] Le K.A., Faeh D., Stettler R., Ith M., Kreis R., Vermathen P., Boesch C., Ravussin E., Tappy L. (2006). A 4-wk high-fructose diet alters lipid metabolism without affecting insulin sensitivity or ectopic lipids in healthy humans. Am. J. Clin. Nutr..

[30] Ngo Sock E.T., Le K.-A., Ith M., Kreis R., Boesch C., Tappy L. (2010). Effects of a short-term overfeeding with fructose or glucose in healthy young males. Brit. J. Nutr..

[31] Stanhope K.L., Bremer A.A., Medici V., Nakajima K., Ito Y., Nakano T., Chen G., Fong T.H., Lee V., Menorca R.I. (2011). Consumption of fructose and high fructose corn syrup increase postprandial triglycerides, LDL-cholesterol, and apolipoprotein-B in young men and women. J. Clin. Endocrinol. Metab..

[32] Stanhope K.L., Schwarz J.M., Keim N.L., Griffen S.C., Bremer A.A., Graham J.L., Hatcher B., Cox C.L., Dyachenko A., Zhang W. (2009). Consuming fructose-sweetened, not glucose-sweetened, beverages increases visceral adiposity and lipids and decreases insulin sensitivity in overweight/obese humans. J. Clin. Investig..

